# Randomized controlled trial of an internet-based self-guided hand exercise program to improve hand function in people with systemic sclerosis: the Scleroderma Patient-centered Intervention Network Hand Exercise Program (SPIN-HAND) trial

**DOI:** 10.1186/s13063-022-06923-4

**Published:** 2022-12-12

**Authors:** Linda Kwakkenbos, Marie-Eve Carrier, Joep Welling, Brooke Levis, Alexander W. Levis, Maureen Sauve, Kimberly A. Turner, Lydia Tao, Kylene Aguila, Andrea Carboni-Jiménez, Mara Cañedo-Ayala, Sami Harb, Cornelia van den Ende, Marie Hudson, Ward van Breda, Christelle Nguyen, Isabelle Boutron, François Rannou, Brett D. Thombs, Luc Mouthon, on behalf of the SPIN Investigators, Richard S. Henry, Susan J. Bartlett, Catherine Fortuné, Karen Gottesman, Geneviève Guillot, Laura K. Hummers, Amanda Lawrie-Jones, Vanessa L. Malcarne, Maureen D. Mayes, Warren R. Nielson, Michelle Richard, Shervin Assassi, Andrea Benedetti, Ghassan El-Baalbaki, Carolyn Ells, Kim Fligelstone, Tracy Frech, Amy Gietzen, Daphna Harel, Monique Hinchcliff, Sindhu R. Johnson, Maggie Larche, Catarina Leite, Karen Nielsen, Janet Pope, Tatiana Sofia, Anne A. Schouffoer, Maria E. Suarez-Almazor, Christian Agard, Nassim Ait Abdallah, Marc André, Elana J. Bernstein, Sabine Berthier, Lyne Bissonnette, Alessandra Bruns, Patricia Carreira, Marion Casadevall, Benjamin Chaigne, Lorinda Chung, Benjamin Crichi, Christopher Denton, Robyn Domsic, James V. Dunne, Bertrand Dunogue, Regina Fare, Dominique Farge-Bancel, Paul R. Fortin, Jessica Gordon, Brigitte Granel-Rey, Aurélien Guffroy, Genevieve Gyger, Eric Hachulla, Ariane L. Herrick, Sabrina Hoa, Alena Ikic, Niall Jones, Suzanne Kafaja, Nader Khalidi, Marc Lambert, David Launay, Yvonne C. Lee, Hélène Maillard, Nancy Maltez, Joanne Manning, Isabelle Marie, Maria Martin Lopez, Thierry Martin, Ariel Masetto, François Maurier, Arsene Mekinian, Sheila Melchor Díaz, Mandana Nikpour, Louis Olagne, Vincent Poindron, Susanna Proudman, Alexis Régent, Sébastien Rivière, David Robinson, Esther Rodríguez, Sophie Roux, Perrine Smets, Vincent Sobanski, Robert Spiera, Virginia Steen, Evelyn Sutton, Carter Thorne, John Varga, Pearce Wilcox, Marie-Nicole Discepola, Laury Montemurro, Elsa Lynn Nassar, Marieke Alexandra Neyer, Julia Nordlund, Nora Østbø, Sabrina Provencher

**Affiliations:** 1 Clinical Psychology, Radboud University, Nijmegen, The Netherlands; 2 Department of Medical Psychology, Radboud Institute for Health Sciences, Radboud University Medical Center, Nijmegen, The Netherlands; 3 Lady Davis Institute for Medical Research of the Jewish General Hospital, 3755 Cote Ste Catherine Road, Montreal, QC H3T 1E2 Canada; 4 NVLE Dutch Patient Organization for Systemic Autoimmune Diseases, Utrecht, The Netherlands; 5 Centre for Prognosis Research, School of Medicine, Keele University, Staffordshire, UK; 6 Department of Biostatistics, Harvard T. H. Chan School of Public Health, Cambridge, MA USA; 7 Scleroderma Society of Ontario, Hamilton, ON Canada; 8 Scleroderma Canada, Hamilton, ON Canada; 9 Sint Maartenskliniek, Nijmegen, The Netherlands; 10 Department of Medicine, McGill University, Montreal, QC Canada; 11 Faculty of Behavioural and Movement Sciences, Vrije Universiteit, Amsterdam, The Netherlands; 12 Université Paris Descartes, Université de Paris, Paris, France; 13 Assistance Publique - Hôpitaux de Paris, Paris, France; 14 Department of Psychiatry, McGill University, Montreal, QC Canada; 15 Department of Epidemiology, Biostatistics, and Occupational Health, McGill University, Montreal, QC Canada; 16 Department of Psychology, McGill University, Montreal, QC Canada; 17 Biomedical Ethics Unit, McGill University, Montreal, QC Canada; 18 Service de Médecine Interne, Centre de Référence Maladies Autoimmunes Systémiques Rares d’Ile de France, Hôpital Cochin, Paris, France

**Keywords:** Cohort multiple RCT, Occupational therapy, Physical therapy, Randomized controlled trial, Scleroderma, Systemic, Systemic sclerosis, Tele-rehabilitation

## Abstract

**Background:**

Systemic sclerosis (scleroderma; SSc) is a rare autoimmune connective tissue disease. Functional impairment of hands is common. The Scleroderma Patient-centered Intervention Network (SPIN)-HAND trial compared effects of offering access to an online self-guided hand exercise program to usual care on hand function (primary) and functional health outcomes (secondary) in people with SSc with at least mild hand function limitations.

**Methods:**

The pragmatic, two-arm, parallel-group cohort multiple randomized controlled trial was embedded in the SPIN Cohort. Cohort participants with Cochin Hand Function Scale (CHFS) scores ≥ 3 and who indicated interest in using the SPIN-HAND Program were randomized (3:2 ratio) to an offer of program access or to usual care (targeted *N* = 586). The SPIN-HAND program consists of 4 modules that address (1) thumb flexibility and strength; (2) finger bending; (3) finger extension; and (4) wrist flexibility and strength. The primary outcome analysis compared CHFS scores 3 months post-randomization between participants offered versus not offered the program. Secondary outcomes were CHFS scores 6 months post-randomization and functional health outcomes (Patient-Reported Outcomes Measurement Information System profile version 2.0 domain scores) 3 and 6 months post-randomization.

**Results:**

In total, 466 participants were randomized to intervention offer (*N* = 280) or usual care (*N* = 186). Of 280 participants offered the intervention, 170 (61%) consented to access the program. Of these, 117 (69%) viewed at least one hand exercise instruction video and 77 (45%) logged into the program website at least 3 times. In intent-to-treat analyses, CHFS scores were 1.2 points lower (95% CI − 2.8 to 0.3) for intervention compared to usual care 3 months post-randomization and 0.1 points lower (95% CI − 1.8 to 1.6 points) 6 months post-randomization. There were no statistically significant differences in other outcomes.

**Conclusion:**

The offer to use the SPIN-HAND Program did not improve hand function. Low offer uptake, program access, and minimal usage among those who accessed the program limited our ability to determine if using the program would improve function. To improve engagement, the program could be tested in a group format or as a resource to support care provided by a physical or occupational therapist.

**Trial registration:**

NCT03419208. Registered on February 1, 2018.

**Supplementary Information:**

The online version contains supplementary material available at 10.1186/s13063-022-06923-4.

## Introduction

Systemic sclerosis (SSc, or scleroderma) is a rare systemic autoimmune disease characterized by vascular hyperreactivity and fibrosis of the skin and internal organs [1, 2]. Approximately 90% of people with SSc experience significant functional limitations in the hands, and hand function is more closely associated with disability than any other aspect of the disease [3–8].

In the absence of a cure, a primary goal of SSc care is to prevent and reduce disability and improve health-related quality of life [1]. Physical and occupational therapy techniques for the hands that have been described include rehabilitation exercises, connective tissue massages, joint manipulation, splinting, and heat or paraffin wax baths [9–14]. Existing evidence, however, tends to be descriptive or from very small trials with important limitations in design, conduct, and reporting [15]. As part of an ongoing living systematic review [16], we identified 11 randomized controlled trials (RCTs) that evaluated physical or occupational therapy interventions designed specifically to improve hand function in SSc, but none analyzed data from ≥ 30 participants per trial arm [13, 14, 17–25]. Two other small trials have evaluated a 12-week multidisciplinary day treatment program (*N* = 53) [26] and a minimally supervised general home exercise program (*N*= 44) [11]; both included hand exercises and reported that the interventions improved grip strength. A large, well-conducted RCT (*N*= 218) tested a 1-month supervised SSc hospital-based exercise therapy program (three weekly sessions of 3 h each for 4 weeks plus personalized home program), which included hand exercises, and reported that the program reduced hand disability at 1-month follow-up (3.7 points on Cochin Hand Function Scale (CHFS), 95% confidence interval [CI] 1.2 to 6.1), but not after 6 or 12 months post-randomization [27]. Current guidelines for SSc management note the potential importance of rehabilitation interventions, but do not recommend for or against them due to limited evidence [28, 29].

Electronic health interventions are increasingly used to address a range of health care problems [30–34] and may be particularly important in rare diseases, such as SSc, where people face barriers to accessing specialized services. The Scleroderma Patient-centered Intervention Network (SPIN) is an international network of researchers, clinicians, and people with SSc [35] that developed an online hand exercise program designed to improve hand function (SPIN-HAND) [36]. Prior to conducting a full-scale RCT, we conducted a randomized feasibility trial (*N* = 40) [36, 37] in which we utilized the cohort multiple randomized controlled trial (cmRCT) design [35, 38] Based on routine cohort assessments, eligible participants were randomized to be offered the SPIN-HAND intervention or to usual care. We found that trial methodology was feasibly implemented and that the online SPIN-HAND Program was acceptable to patients, but uptake of the offer and program usage were low [37]. To attempt to address the low offer acceptance, in designing the full-scale trial we evaluated the interest of potential participants in using the intervention as part of trial eligibility criteria.

The aim of the full-scale SPIN-HAND trial was to evaluate the effect of being offered access to the SPIN-HAND Program, compared to usual care alone, on hand function (primary) and functional health outcomes (secondary).

## Methods

The SPIN-HAND trial was a pragmatic, two-arm parallel cmRCT with a 3:2 allocation ratio to an offer of access to the SPIN-HAND Program in addition to usual care or to usual care alone. The trial was embedded in the SPIN Cohort and registered prior to enrolling participants (NCT03419208). Ethics approval was obtained from the Research Ethics Committee of the Jewish General Hospital, Montreal, Canada (#2013–147, 12–123-A).

### SPIN Cohort

The SPIN Cohort was developed as a framework for embedded pragmatic trials using the cmRCT design [35, 38]. Participants in the SPIN Cohort enroll in an observational cohort with regular outcome measurement and consent to (1) allow their data to be used for observational studies; (2) allow their data to be used to assess intervention trial eligibility and, if eligible, be randomized; (3) if randomized to the intervention arm of a trial to be contacted and invited to consent to access and use an intervention; and (4) if randomized to usual care, allow their data to be used to evaluate intervention effectiveness without being notified that they are in the trial control group [35].

To be eligible for the SPIN Cohort, participants must be classified as having SSc based on 2013 American College of Rheumatology / European League Against Rheumatism criteria [39], confirmed by a SPIN physician; be ≥ 18 years old; be fluent in English, French or Spanish; and be able to respond to questionnaires via the internet. The SPIN Cohort is a convenience sample. Participants are recruited at SPIN sites (www.spinsclero.com/en/sites) during regular medical visits, and written informed consent is obtained. A medical data form is submitted online by the site to enroll participants. Cohort participants complete outcome measures via the internet upon enrollment and subsequently every 3 months [35]. SPIN Cohort enrollment started in March 2014 and is ongoing. The SPIN Cohort was approved by the Research Ethics Committee of the Jewish General Hospital, Montréal, Canada (#12–123), and by the research ethics committees of each recruiting center.

Among sites with participants in the SPIN Cohort at the start of the trial, 32 sites from Canada, France, the USA, the UK, and Australia were included in the trial (81% of active participants); however, the Australian site had just started patient enrollment. Two sites in Spain and Mexico were excluded because the SPIN-HAND Program was not available in Spanish; participants from 3 sites were excluded due to concerns about other concurrent non-SPIN activities at those sites; and participants from 4 sites were excluded because they were involved in a concurrent feasibility trial of SPIN’s self-management program (SPIN-SELF) [40]. These 4 sites were from English-speaking countries or regions and were chosen based on the number of participants they had enrolled that would yield sufficient numbers for the SPIN-SELF feasibility trial.

### SPIN-HAND trial eligibility

Assessment of trial eligibility occurred during participants’ regular SPIN Cohort assessments. Cohort participants were eligible for the SPIN-HAND trial if they completed their SPIN Cohort measures in English or French, reported at least mild hand function limitations (CHFS [41] ≥ 3), and indicated interest in using an online hand exercise intervention (≥ 6 on 0–10 scale). SPIN Cohort participants who met these criteria were then shown a webpage that described that SPIN was planning to do a study to evaluate the effect of an online program of hand exercises to prevent or reduce hand function problems in people with SSc, provided a description of the SPIN-HAND Program with screenshots of the most important features (i.e., program modules, exercise videos, goal setting, and patient stories) and the time required from participants, and asked whether, if invited, they would be willing to try the program (yes/no); those who answered “yes” were eligible and randomized. Participants randomized to the intervention group in the SPIN-HAND feasibility trial [36, 37] were excluded from the full-scale trial, but participants in the feasibility trial control arm were eligible because they had not been notified about the feasibility trial and did not have access to the SPIN-HAND Program during the feasibility trial.

### Procedure: randomization, allocation concealment, consent, and blinding

Eligible participants were randomized automatically as they completed their regular SPIN Cohort assessments using a feature in the SPIN Cohort platform, which provided immediate centralized randomization and complete allocation sequence concealment. Participants randomized to usual care were not notified about the trial and completed their regular SPIN Cohort assessments [35, 38]. Thus, participants who were offered the intervention were not blind to their status, whereas participants assigned to usual care were blind to their participation in the trial and assignment to usual care. We used a 3:2 intervention to usual care control allocation ratio to be able to potentially evaluate dose–response effects as we anticipated that some intervention participants would not consent to use the program.

Participants randomized to the intervention arm received an automated email invitation including the brief screencast video about program features, a link to the program website, and a copy of the intervention consent form. At initial login to the SPIN-HAND platform, they were prompted to consent to participate, and those who consented were re-directed to the program introduction page. Participants who logged out without consenting returned to the consent page upon subsequent logins, if any.

SPIN personnel attempted to contact all intervention arm participants by phone, usually within 48 h of sending the invitation email, to describe the study, review the consent form, and answer questions. If participants could not be reached initially, up to 5 attempts were made in the 10 days post-randomization. If a participant was unreachable after five attempts, a sixth and last attempt was conducted approximately 20 days post-invitation. Email and phone technical support were available to help participants with the consent process and to access and use the intervention site.

### Intervention and comparator

#### SPIN-HAND Program

Intervention arm participants had access to the SPIN-HAND Program through any electronical device (e.g., computer, tablet, smartphone) for the entire 6-month trial period. The program was designed by SPIN experts and members of the SPIN Patient Advisory Board [36]. The program utilizes exercises from rehabilitation programs that have improved hand function in SSc [27] and rheumatoid arthritis [42] and integrates key components of successful disease self-management programs, including goal setting and feedback, social modeling, and mastery experiences [43–47]. The core of the program consists of 4 modules that address (1) thumb flexibility and strength, (2) finger bending, (3) finger extension, and (4) wrist flexibility and strength.

The program includes sections on developing a personalized program, goal-setting strategies, progress tracking, sharing goals with friends or family, and patient stories about hand disability and exercises. Instructional videos explain and demonstrate how to perform each exercise properly with pictures to illustrate common mistakes. Participants can select modules in any order, based on a description of the type of function that the module targets. They are provided guidance on selecting exercise intensity levels by screenshots and descriptions of mild to moderate and severe hand involvement. Separate versions of exercises and instructional videos are available for both involvement levels. Additionally, some exercise videos for participants with severe hand involvement are complemented with pictures illustrating alternate versions of the exercise if the original exercise cannot be performed.

For the first 4 weeks of the program, participants focus on exercises in one module per week and are encouraged to perform the exercises 3–5 times per week. Time per day graduates from 3–4 min in week 1 to 5–15 min in week 4. Starting with week 5, participants can select from a menu of program options that fit their needs and schedule. These range from 5–10 min per day to 30–35 min per day.

The SPIN-HAND Program can be accessed online at https://tools.spinsclero.com.

#### Comparator

Participants in the usual care arm received usual care and routine reminders to complete regular SPIN Cohort measures. They did not have access to the SPIN-HAND Program and were not alerted that they were participants in the trial.

There were no restrictions on participants in the intervention or comparator arms with respect to any other hand-related interventions that might have been part of usual care outside of the trial.

### Outcome measures

The primary outcome was CHFS score 3 months post-randomization [41]. Secondary outcomes were CHFS score 6 months post-randomization and patient-reported functional health outcomes measured with the Patient-Reported Outcomes Measurement Information System (PROMIS-29) profile version 2.0 [48] at 3 and 6 months post-randomization. These trial outcome measures are routinely assessed as part of SPIN Cohort assessments. Additionally, the Client Satisfaction Questionnaire-8 (CSQ-8) [49] was administered to intervention arm participants to assess program satisfaction.

Participants reported sociodemographic information via the SPIN Cohort. SPIN physicians provided medical information upon enrollment of participants in the SPIN Cohort, including dates of initial onset of Raynaud’s phenomenon symptoms, non-Raynaud phenomenon symptoms, and diagnosis; SSc subtype (limited, diffuse, sine); presence of joint contractures in small and large joints (none; mild [≤ 25% range of motion limitation]; moderate to severe [> 25%]); presence of tendon friction rubs (currently present or absent); and modified Rodnan Skin Score (mRSS) [50].

The 18-item CHFS [41] measures the ability to perform hand-related activities. Items are scored on a 0–5 Likert scale (0 = *without difficulty;* 5 = *impossible*). The total score is obtained by adding the scores of all items (range 0–90). Higher scores indicate less functionality. The CHFS has good convergent validity with general functional disability measures and good sensitivity to change [41, 51, 52]. It has been validated in SSc [52].

The PROMIS-29v2.0 measures health status with 4 items each for the domains of physical function, anxiety, depression, fatigue, sleep disturbance, social roles and activities, and pain interference, plus a single item for pain intensity [48]. Items in domains are scored on a 5-point scale (item score range 1–5), and pain intensity is measured on an 11-point rating scale. Higher scores represent more of the domain being measured (i.e., better physical function and ability to participate in social roles and activities, but higher symptom levels). The sum of item scores for each domain are converted into *T*-scores standardized from the general US population (mean = 50, standard deviation [SD] = 10). The PROMIS-29v2.0 has been validated in SSc [53, 54].

The 8-item CSQ-8 measures client satisfaction with health care services. Items were modified slightly to refer to the SPIN-HAND Program, as opposed to a generic service. Items are scored on a 4-point Likert scale (1 = *low satisfaction*, 4 = *high satisfaction*). Total scores range from 8 to 32, with higher scores indicating higher satisfaction [49].

Logins to the SPIN-HAND Program and usage of program components were assessed using automated usage logs collected through the SPIN-HAND platform.

### Sample size

A standardized mean difference effect size of 0.25 may represent a clinically important difference in hand function for rheumatic disease patients [55]. Trials of home exercise programs in SSc [27] and rheumatoid arthritis [42] reported effect sizes of approximately 0.40 and 0.30, respectively. For an assumed effect size of standardized mean difference (SMD) = 0.25, two-tailed with *α* = 0.05, and 3:2 randomization, data from *N* ≥ 528 participants would have provided 80% power. Assuming 10% loss to follow-up, our target sample size was to randomize 586 patients.

### Statistical analyses

See Supplementary Material for the full statistical analysis plan. All analyses were conducted in R (R version 3.6.3; R Studio version 1.2.5042). We used an intent-to-treat analysis to estimate differences in scores between intervention and control participants with a linear mixed effects model fit using the lme function in nlme [56]. Intervention effects were adjusted for baseline CHFS scores, sex, age, disease duration, and diffuse versus limited SSc as fixed effects. To account for the different enrollment centers, we fit a random intercept for each site. Score differences were presented with 95% CIs.

To minimize the possibility of bias from missing outcome data, we used multiple imputation by chained equations using the mice package to generate 20 imputed datasets, using 15 cycles per imputed dataset. Variables in the mice procedure included center of enrolment, intervention arm, consent, measures of all primary and secondary outcomes at all three timepoints, age, sex, subtype, years since onset of the first non-Raynaud’s phenomenon symptom, presence of small joint contractures, and patient-reported severity of Raynaud’s phenomenon symptoms (0–10 scale) and digital ulcers (0–10 scale) at baseline. Pooled standard errors and associated 95% CIs were estimated using Rubin’s rules [57].

To estimate average intervention effects among compliers (defined as consent to use the SPIN-HAND Program), we used an instrumental variable approach [58, 59] to inflate intention-to-treat effects from main models by the inverse probability of compliance among intervention arm participants; 95% CIs were constructed via bootstrap with resampling [60, 61].

Statistical analyses were done blind to trial arm allocation, except for the complier effect analyses, which required knowledge of intervention arm consent. All analyses were 2-sided with *α* = 0.05. The frequency of logins and number of times modules and exercise videos were accessed were calculated from the usage log data. Usage data and the satisfaction outcome were reported using descriptive statistics.

### Protocol amendments

There were two amendments to the trial protocol. First, initially, the EuroQoL-5D-5L [62] was specified as a secondary outcome in the trial registration. This outcome was removed prior to collecting outcome data. It was initially included to compute quality-adjusted life years for economic analyses. However, items of the EuroQoL-5D-5L do not align with SPIN-HAND Program targets, so it was not included in the trial, but the trial registration was not adjusted. Second, because we did not find evidence of improved hand function at 3 or 6 months post-randomization, and it is not plausible that there will be delayed effects given the low intervention uptake, we will not conduct planned analyses at 12 and 24 months post-randomization.

### Patient and public involvement

Members of the SPIN Patient Advisory Board participated in the conception and development of the SPIN-HAND Program, design of the trial, selection of primary and secondary trial outcomes, development of trial procedures, and interpretation of study results. One of the members worked directly with researchers on the intervention development team to film the hand exercises and edit videos for the online program. All members reviewed and commented on this document.

### Role of the funding source

Funders had no role in any aspect of study design; data collection, analysis, and interpretation; manuscript drafting; or the decision to submit for publication.

## Results

### Participants

Enrollment started on March 12, 2018, and ended on November 19, 2019. During the period of enrollment, there were 1955 active SPIN Cohort participants, of whom 1592 were from sites included in the SPIN-HAND trial. A total of 1293 participants completed the eligibility forms as part of their regular assessments at least once during the trial period and were assessed for eligibility. Of these, 496 met inclusion criteria based on the CHFS and signalling item criteria. Thirty (6%) of these answered “no” to the question of whether they would try the SPIN-HAND Program if offered to them and were excluded. Thus, 466 (36%) of participants who were assessed met all inclusion criteria and were randomized. Enrollment was stopped before reaching the targeted sample size of 586 participants because all Cohort participants had had at least two opportunities to complete Cohort assessments, and enrollment of a substantial number of additional participants was unlikely.

**Fig. 1:**
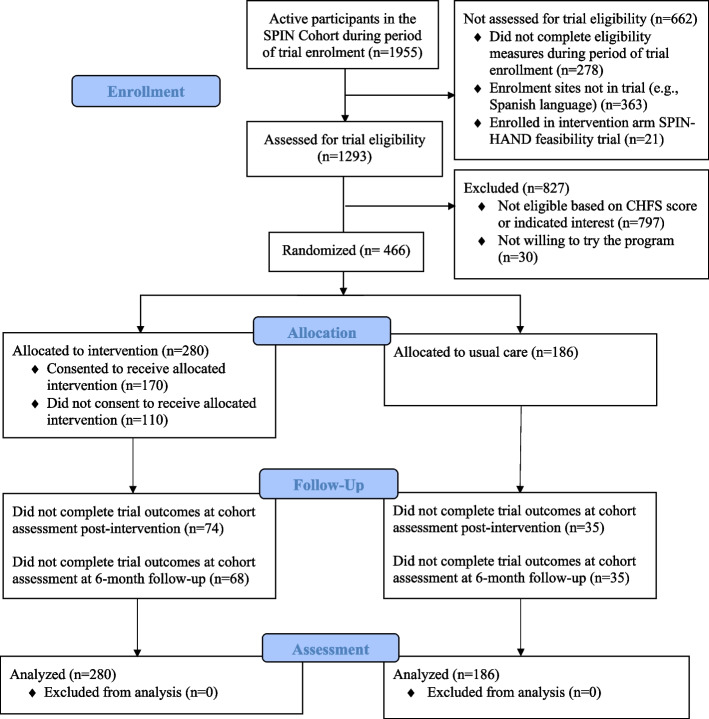
Flow diagram for the SPIN-HAND trial

Of the 466 participants, 280 (60%) were allocated to the intervention, and 186 (40%) to usual care. See Fig. 1 for SPIN Cohort participant flow through the trial. As shown in Table 1, intervention and control participants were similar. Overall, mean age was 55.8 years (SD = 12.4), 90% (*N* = 418) were female, and 87% (*N* = 396) identified as White. Participants were from the USA (*N* = 167, 36%), Canada (*N* = 125, 27%), France (*N* = 103, 22%), the UK (*N* = 70, 15%), and Australia (*N* = 1, < 1%). Mean time since diagnosis was 11.0 years (SD = 7.7 years), and 46% (*N* = 212) of participants had diffuse SSc. Upon enrolment in the trial, 93 of 280 (33%) and 55 of 186 (30%) of participants indicated having physical or occupational therapy in the previous 3 months (35 and 29% at 3 months follow-up, respectively). In total, 170 of 280 (61%) participants randomized to be offered the SPIN-HAND intervention consented to access the program. CHFS scores were similar for participants assigned to the intervention who consented to access the program (mean = 22.5, SD = 17.1) and those who did not (mean = 21.5, SD = 16.5).

**Table 1:** Participant characteristics

Variable	Intervention *N* = 280	Usual care *N* = 186
**Demographic**		
Age in years	56.0 (12.7)	55.4 (11.9)
Female sex, *N* (%)	250 (89.3%)	168 (90.3%)
Education in years	15.6 (3.6)^a^	15.7 (3.5)^b^
Married or living as married, *N* (%)	186 (68.4%)^a^	128 (69.2%)^b^
Race/ethnicity, *N* (%)		
White	236 (86.8%)^a^	160 (86.5%)^b^
Black	19 (7.0%)^a^	12 (6.5%)^b^
Other	17 (6.3%)^a^	13 (7.0%)^b^
Country, *N* (%)		
Canada	77 (27.5%)	48 (25.8%)
United States	105 (37.5%)	62 (33.3%)
France	64 (22.9%)	39 (21.0%)
United Kingdom	33 (11.8%)	37 (19.9%)
Australia	1 (0.4%)	0 (0.0%)
Physical or occupational therapy, *N*(%)	93 (33.2%)	55 (29.6%)
**Disease characteristics**		
Time since onset first non-Raynaud’s symptoms in years	13.1 (8.7)^c^	11.7 (7.7)^d^
Time since onset Raynaud’s in years	16.6 (12.0)^e^	14.6 (10.1)^f^
Time since diagnosis in years	11.4 (8.1)^g^	10.4 (6.9)^h^
Diffuse disease subtype, *N* (%)^i^	127 (46.0%)^j^	85 (46.7%)^k^
Modified Rodnan Skin Score^i^	9.9 (9.3)^l^	9.8 (9.3)^m^
Small joints contractures, *N* (% positive)^i^	77 (30.2%)^n^	67 (37.9%)^o^
Large joint contractures, *N* (% positive)^i^	43 (17.3%)^p^	28 (16.2%)^q^
Tendon friction rubs currently, *N* (% positive)^i^	31 (13.3%)^r^	17 (11.2%)^s^
**Cochin Hand Function Scale**		
CHFS score	22.1 (16.8)	23.3 (16.7)
**PROMIS-29v2 domains**		
Physical function score	40.0 (7.5)^t^	40.7 (7.8)^u^
Anxiety symptom score	54.0 (10.4)^t^	54.6 (9.8)^v^
Depression symptom score	53.5 (9.3)^t^	53.2 (9.5)^v^
Fatigue score	58.4 (10.2)^t^	57.0 (10.8)^v^
Sleep disturbance score	54.0 (8.9)^t^	53.0 (7.4)^v^
Social roles and activities score	44.9 (8.8)^t^	44.9 (9.2)^v^
Pain interference score	57.6 (8.9)^w^	59.0 (8.8)^v^
Single item for pain intensity	4.8 (2.5)^w^	4.4 (2.5)^k^

Mean (SD) are reported unless indicated

*N* number, *SD* standard deviation

Due to missing data: ^a^*N* = 272; ^b^*N* = 185; ^c^*N* = 261; ^d^*N* = 167; ^e^*N* = 242; ^f^*N* = 166; ^g^*N* = 269; ^h^*N* = 178; ^i^Assessed at time of enrollment in the SPIN Cohort (mean time to enrolment in trial = 2.2 years (SD = 1.3); ^j^*N* = 276; ^k^*N* = 182; ^l^*N* = 213; ^m^*N* = 141; ^n^*N* = 255; ^o^*N* = 177; ^p^*N* = 248; ^q^*N* = 173; ^r^*N* = 234; ^s^*N* = 152; ^t^*N* = 279; ^u^*N* = 184; ^v^*N* = 183; ^w^*N* = 278

### Usage of the SPIN-HAND Program

Between the baseline and 3-month assessments, 175 of the 280 (63%) participants randomized to the intervention logged in to the online intervention website, and 170 (61%) consented to use the program, though one person who consented did so after the 3-month follow-up.

During the trial period, of the 170 participants who consented, 48 (28%) logged in only once, 45 (26%) logged in twice, 50 logged in three or four times (29%), and 27 (16%) logged in more than 4 times (range 5 to 42 times). Overall, the mean number of logins was 3.8 (SD = 5.3; median = 2). Eighty-one participants (48%) used the website tour, and 117 (69%) viewed at least one hand exercise video in any of the 4 modules; 44 participants (26%) accessed 1 of the 4 modules, 23 (14%) accessed 2 modules, 16 (9%) accessed 3 modules, and 34 participants (20%) accessed all 4 modules. Among the modules, 92 participants (54%) accessed the Thumb Flexibility and Strength module, 71 (42%) accessed the Finger Bending module, 61 (36%) accessed the Finger Extension module, and 50 (29%) accessed the Wrist Flexibility and Strength module. The goal-setting feature was used by 46 participants (27%), and 4 participants (2%) shared goals with friends or family.

### Trial outcomes

Outcome data were obtained for 357 of 466 (77%) participants at 3 months post-randomization, including 74% (206 of 280) of intervention participants and 81% (151 of 186) of usual care participants. At 6 months post-randomization, 363 (78%) participants completed at least one outcome measure in their SPIN Cohort assessment, including 212 of 280 (76%) intervention and 151 of 186 (81%) usual care participants. Table 2 shows complete-data outcomes at each time point.

**Table 2:** Outcome data immediately post-intervention and 6 months post-randomization (complete data only)

Variable	Intervention	Usual Care
**Post-intervention (intervention *N* = 206; usual care *N* = 151):**		
**Cochin Hand Function Scale** score	20.2 (16.6)	23.0 (18.3)^a^
**PROMIS-29v2 domains**		
Physical function score	40.7 (7.8)	41.1 (7.9)
Anxiety symptom score	53.6 (10.5)	53.7 (10.2)
Depression symptom score	52.3 (9.8)	52.5 (10.0)
Fatigue score	57.7 (8.9)	56.7 (11.1)
Sleep disturbance score	53.2 (8.3)^b^	52.7 (8.3)
Social roles and activities score	45.3 (8.5)	45.9 (9.2)
Pain interference score	58.4 (8.6)	57.1 (9.8)
Single item for pain intensity	4.8 (2.5)	4.2 (2.6)^c^
**6 months post-randomization (intervention *N* = 212; usual care *N* = 151):**		
**Cochin Hand Function Scale** score	21.1 (16.4)^d^	22.0 (19.0)^a^
**PROMIS-29v2 domains**		
Physical function score	40.7 (7.6)	41.0 (8.4)
Anxiety symptom score	53.5 (10.4)	53.7 (10.2)
Depression symptom score	52.0 (9.6)	52.6 (10.0)
Fatigue score	57.6 (9.4)^e^	55.4 (11.2)
Sleep disturbance score	54.1 (7.9)	52.4 (8.0)
Social roles and activities score	45.3 (8.7)	46.3 (10.0)
Pain interference score	58.1 (8.6)	56.7 (9.7)
Single item for pain intensity	4.7 (2.4)	4.1 (2.6)^c^

Mean (SD) are reported unless indicated

*N* number, *SD* standard deviation Due to missing values ^a^*N* = 149; ^b^*N* = 205; ^c^*N* = 150; ^d^*N* = 2019; ^e^*N* = 211

As shown in Table 3, in the primary intent-to-treat analysis, CHFS scores were not statistically significantly different between groups immediately post-intervention. Post-intervention CHFS scores were 1.2 points lower (95% CI 2.8 points lower to 0.3 points higher) for intervention compared to usual care. In average complier effect analysis, this difference was 2.0 points lower (95% CI 4.7 points lower to 0.7 points higher). At 6 months post-randomization, results were similar between intervention and usual care (− 0.1 points, 95% CI − 1.8 to 1.6 points), including in average complier effect analysis (− 0.1 points, 95% CI − 3.1 to 2.9 points). Among other secondary outcomes, none of the PROMIS-29v2 domain scores were statistically significantly different immediately post-intervention or 6 months post-randomization. Results from complete case analyses are provided in the Supplementary Material.

**Table 3:** Trial outcomes: intention to treat and estimated average complier effect

	**Intention to treat**	**Estimated average complier effect**
	**Difference (95% CI)**	**Difference (95% CI)**
**Primary outcome (post-intervention):**		
Cochin Hand Function Scale	− 1.24 (− 2.77 to 0.28)	− 2.04 (− 4.73 to 0.66)
**Secondary outcomes (post-intervention):**		
PROMIS-29v2 Physical function	0.39 (− 0.44 to 1.23)	0.64 (− 1.00 to 2.28)
PROMIS-29v2 Anxiety symptoms	− 0.03 (− 1.51 to 1.45)	− 0.05 (− 2.62 to 2.53)
PROMIS-29v2 Depression symptoms	− 0.85 (− 2.20 to 0.51)	− 1.38 (− 3.79 to 1.03)
PROMIS-29v2 Fatigue	− 0.27 (− 1.63 to 1.09)	− 0.44 (− 2.87 to 1.99)
PROMIS-29v2 Sleep disturbance	0.26 (− 1.07 to 1.59)	0.43 (− 1.97 to 2.83)
PROMIS-29v2 Social roles and activities	− 0.20 (− 1.39 to 0.99)	− 0.33 (− 2.54 to 1.88)
PROMIS-29v2 Pain interference	− 0.13 (− 1.40 to 1.15)	− 0.21 (− 2.59 to 2.17)
PROMIS-29v2 Single item for pain intensity	0.13 (− 0.22 to 0.48)	0.21 (− 0.64 to 1.06)
**Secondary outcomes (6 months post-randomization):**		
Cochin Hand Function Scale	− 0.09 (− 1.81 to 1.64)	− 0.14 (− 3.13 to 2.85)
PROMIS-29v2 Physical function	0.42 (− 0.48 to 1.32)	0.69 (− 1.05 to 2.43)
PROMIS-29v2 Anxiety symptoms	− 0.03 (− 1.55 to 1.49)	− 0.05 (− 2.68 to 2.59)
PROMIS-29v2 Depression symptoms	− 1.19 (− 2.59 to 0.22)	− 1.94 (− 4.47 to 0.59)
PROMIS-29v2 Fatigue	0.62 (− 0.78 to 2.02)	1.01 (− 1.51 to 3.52)
PROMIS-29v2 Sleep disturbance	1.12 (− 0.09 to 2.43)	1.84 (− 0.42 to 4.10)
PROMIS-29v2 Social roles and activities	− 0.51 (− 1.77 to 0.74)	− 0.84 (− 3.07 to 1.39)
PROMIS-29v2 Pain interference	− 0.15 (− 1.46 to 1.16)	− 0.25 (− 2.59 to 2.10)
PROMIS-29v2 Single item for pain intensity	0.05 (− 0.32 to 0.41)	0.08 (− 0.80 to 0.95)

*CI* confidence interval

### Satisfaction with the SPIN-HAND Program

Among intervention participants who completed the CSQ-8 post-intervention (*N* = 122), satisfaction with the program was moderately high. The mean total score was 25.5 (SD = 4.1). Mean item score was 3.2 on a 1 to 4 scale. See Table 4. The mean score was similar for participants who logged in more than once (*N* = 87; mean = 25.9, SD = 4.2) than for those who logged in only once (*N* = 34; mean = 24.3, SD = 3.8).

**Table 4:** Mean item and total scores for Client Satisfaction Questionnaire-8 (*N* = 122)

Item	Mean (SD)
1. How would you rate the quality of the SPIN-HAND Program?	3.2 (0.7)
2. Did the SPIN-HAND Program provide you the kind of experience you wanted?	3.1 (0.6)
3. To what extent has the SPIN-HAND Program met your needs?	2.8 (0.6)
4. If a friend were in need of similar help, would you recommend the SPIN-HAND Program to him/her?	3.6 (0.6)
5. How satisfied are you with the amount of help you received from the SPIN-HAND Program?	3.1 (0.7)
6. Has the SPIN-HAND Program helped you to deal more effectively with your hand problems?	3.1 (0.7)
7. In an overall, general sense, how satisfied are you with the SPIN-HAND Program?	3.2 (0.7)
8. If you were to seek help again, would you come back to the SPIN-HAND Program?	3.4 (0.7)^a^
Total score	25.5 (4.1)^a^

*SD* standard deviation

^a^Due to missing values *N* = 121

## Discussion

We tested whether the offer to access and use the SPIN-HAND online, self-guided hand exercise program would improve hand function and functional health outcomes. We found that no outcomes differed statistically significantly between participants who received the offer and participants assigned to usual care immediately post-intervention or at 6 months post-randomization. Our ability to draw conclusions about potential effectiveness of the SPIN-HAND Program, if used, however, was limited by the low rate of consent to access the intervention among those offered program access (61%) and the low rate of usage among those who did consent (45% logged in 3 or more times).

Based on our SPIN-HAND feasibility trial, it was expected that with approximately 1800 active participants in the SPIN Cohort of whom 36% would meet eligibility criteria, including 586 participants in the full-scale trial was feasible [37]. During the trial period, however, fewer than 1300 unique SPIN Cohort participants were assessed for trial eligibility due to cohort dropout over time and the need to exclude several sites, resulting in the inclusion of fewer participants than targeted.

Of even greater concern, almost 40% of participants in the trial who indicated pre-enrollment they would try the program if offered and were randomized to the intervention group did not consent to try the SPIN-HAND Program. Among those who did consent to use the program, actual usage was very low. Notably, 94% of all otherwise eligible participants indicated they would accept an offer to use the program if selected, suggesting that most people will agree and that this type of query may not be useful for separating potential participants who will consent and use programs like SPIN-HAND.

The cmRCT design is a post-randomization consent trial design [63–65]. In post-randomization consent trial designs with individual randomization (i.e., not cluster designs), randomization is done based on eligibility criteria prior to obtaining consent from participants. In most cmRCT trials [66–68], as with SPIN-HAND, cohort participants consent upon enrollment in the cohort to participate in possible future post-randomization consent trials; but, when such a trial is conducted, only those selected for the intervention, which is added to usual care, are approached to provide informed consent. Rather than evaluating an intervention among those who agree to receive it, these trials evaluate the effects of being offered an intervention [38].

Post-randomization consent or Zelen trials, when originally proposed, were conducted with participants with serious or life-threatening conditions that require treatment (e.g., cancer, neonatal conditions with high mortality risk) [63, 64] and where participants will almost certainly choose to receive some intervention; in these cases, competing interventions were evaluated. Important components of the rationale for using these designs included improving recruitment to trials and reducing expected disappointment effects, including crossover and dropout, among control participants in conventional trial models assigned to a standard treatment [63–65]. Indeed, in early trials done in cancer patients with the Zelen design, acceptance to participate was over 80% in most trials [63]. These designs have also been used effectively in trials that involve prevention or other public health interventions where both uptake and possible intervention benefits must be considered in evaluating intervention effects (e.g., prevention of suicide among high-risk individuals; offers of smoking cessation services to hospitalized smokers) [65, 69, 70]. More recently [27] a post-randomization consent trial compared a supervised, individualized physical therapy program to usual care in SSc, and acceptance of the intervention offer among randomized patients was high: 110/112 (98%) participants consented to the physical therapy program and 86% attended at least one session. This trial involved recruitment by treating physicians and in-person physical therapy that was not available in usual care.

The SPIN-HAND trial and other cmRCT trials [66–68] differ from most, though not all, trials that have successfully used Zelen designs in that they have typically used a post-randomization consent model for testing the offer of an intervention among people not seeking an intervention or in areas like prevention where the effect of combined uptake and effectiveness are of interest. Rather, investigators using the design have generally been interested in evaluating use of the intervention but have randomized participants to an offer of the intervention or no offer. cmRCT trials that have published results have consistently reported acceptance rates of < 55% [66–68]. In the case of SPIN-HAND, we were interested in the effect of using the intervention, but we hoped that the cmRCT design would facilitate enrollment and reduce disappointment bias. We mistakenly assumed that since there are few interventions available in SSc, uptake would be very high, even though SPIN Cohort participants were not specifically seeking help for hand function limitations. An important difference with a previous post-randomization consent trial that compared a supervised, individualized physical therapy program to usual care in SSc [27] was that for that trial, explanations regarding the study and intervention were given by the patient’s physician rather than a computerized procedure with follow-up from a research team, which may explain why acceptance was much higher in that study compared to the SPIN-HAND trial or other cmRCT trials.

A possible solution that has been suggested to reduce non-acceptance of intervention offers in the cmRCT design is to present cohort participants with a list of possible interventions as part of cohort assessments and ask if they would agree to use them if offered [38], as we did in our trial. In addition to this list of possible interventions, based on the low acceptance of the offer in our previous SPIN-HAND feasibility trial (63%) [37], we attempted to increase the acceptance of the SPIN-HAND intervention offer by providing more detailed information and adding another item to assess interest in participating. This additional eligibility criterion, however, did not improve consent in our trial. This suggests that these so-called “signalling items” may not be effective at identifying intervention accepters in advance. We took a similar approach in a recently completed feasibility trial of SPIN’s online self-management program that also enrolled participants through the SPIN Cohort, and results were similar (*N*= 40; consent rate 35%) [71].

In addition to trial design elements, the eligibility threshold for the CHFS (≥ 3 on 90-point scale) was low, and some participants in our SPIN-HAND trial may have had limited motivation to participate as their hand disability was mild. A recent study among 151 patients with SSc reported that the patient acceptable symptom state for CHFS score in people with SSc is 26 [72]. There was no difference in mean scores, however, between those who consented and those who did not, and many participants had high CHFS scores, suggesting this may not have been the central issue.

Another potential reason for the low uptake of the SPIN-HAND Program could be that it was delivered without support from a health professional. We designed the intervention as a self-guided online program to overcome barriers to delivering a rehabilitation intervention to people with a rare disease [35]. Online delivery of interventions is increasingly common and effective for addressing a range of healthcare problems. User adherence to online, self-administered interventions is low across settings, however, including behavioral and exercise interventions [73–75]. Given that the SPIN-HAND Program targets a health concern that is relevant to people with SSc, which is known as a factor that improves engagement [74], and because no alternative disease-specific exercise programs are available for many, we believed the uptake would be higher for this group, but this was not the case. Higher rates of adherence are typically obtained in interventions that include some human contact [74]. It is possible that the SPIN-HAND Program could be used more effectively as a resource in a blended care format, in which people with SSc could work with their local physical or occupational therapist to develop an individually tailored hand exercise program. Consistent with this concern about low uptake in a self-guided context, we have re-designed our SPIN-SELF intervention as a group-based intervention and are implementing a second feasibility trial with the new format and using a conventional parallel groups trial design within the SPIN Cohort rather than the cmRCT [76]. At the beginning of the COVID-19 pandemic, we quickly launched a cohort-based group mental health intervention using a similar strategy; participation was very high, and follow-up data were collected from over 90% of enrolled participants [77].

Clinically, the SPIN-HAND Program could potentially be used as an adjunct to face-to-face physical or occupational therapy, and people with SSc could also use it in its self-guided online-only format, given that we do not believe that harms from using the program are likely. Potential users should be advised, however, that we do not know how much, if any, benefit would be accrued, if used. The SPIN-HAND Program is now available publicly, free-of-charge https://tools.spinsclero.com, and potential users have access to an overview with an explanation of what we know about likely effects of using the program (https://www.spinsclero.com/en/projects/spin-hand-toolkit).

Our trial and others suggest that the use of the cmRCT design may need careful re-consideration. The use of a cohort as an infrastructure for trials does appear to have benefits in terms of reductions in resources needed for multi-site trials and for having a pool of participants for multiple trials. Investigators may thus consider cohort-based trials for efficiency but without the prerandomization consent component of the cmRCT. For future SPIN trials, because of the low uptake of the intervention offer, we have revised our trial design, and instead of the cmRCT design, we will obtain consent for trials and randomize post-consent to interventions or comparators [76].

The present study has limitations that should be considered in interpreting its results. First and foremost, because of the poor consent rate and low usage of the SPIN-HAND Program, we do not know how effective the intervention would be if it were used by people with SSc. Second, the SPIN Cohort constitutes a convenience sample of SSc patients receiving treatment at a SPIN recruiting center and SSc patients in the SPIN Cohort complete questionnaires online which may limit the generalizability of finding as all participants already have Internet access and are comfortable using it in a research setting. A comparison between SPIN Cohort participants and the European Scleroderma Trials and Research (EUSTAR) and Canadian Scleroderma Research Group (CSRG) cohorts, however, showed that the SPIN Cohort is broadly comparable with these cohorts, increasing confidence that insights gained from the SPIN Cohort should be generalizable [78].

## Conclusions

In sum, we did not find a significant effect of our online SPIN-HAND Program on hand function. The low uptake of the offer of access and the limited usage of the SPIN-HAND Program of participants in the intervention arm, however, reduced our ability to draw conclusions about effectiveness if the program were used more actively. For future trials of SPIN interventions, to address the issue of a low uptake of the intervention offer, we have revised our trial design and, instead of the cmRCT design, we will obtain consent for the trial and randomize post-consent as in conventional RCT designs. In addition, we will re-design our interventions as online group-based interventions to improve engagement among participants.

## Supplementary Information

**Additional file 1.** Supplementary material.

## Data Availability

The datasets used and/or analyzed during the current study are available from the corresponding author on reasonable request.

## Abbreviations

CHFS: Cochin Hand Function Scale
cmRCT: Cohort multiple Randomized Controlled Trial
CSQ-8: Client Satisfaction Questionnaire-8
mRSS: Modified Rodnan Skin Score
PROMIS-29: Patient-Reported Outcomes Measurement Information System-29
RCT: Randomized controlled trial
SPIN: Scleroderma Patient-centered Intervention Network
SSc: Systemic sclerosis
