# Supplementary material for: Randomized controlled trial of an internet-based self-guided hand exercise program to improve hand function in people with systemic sclerosis: the Scleroderma Patient-centered Intervention Network Hand Exercise Program (SPIN-HAND) trial
Source: Trials. 2022 Dec 12;23:994. doi: 10.1186/s13063-022-06923-4 (PMC9742661; doi:10.1186/s13063-022-06923-4)
Supplement: Supplementary file 1 — Additional file 1. Supplementary material. [file 13063_2022_6923_MOESM1_ESM.docx]

**Supplementary Material**

**eMethods1.** Statistical analysis plan

**eTable1.** Trial outcomes: complete cases and estimated complier-average causal effect with complete cases

**eMethods1**. Statistical analysis plan

**STATISTICAL ANALYSIS PLAN (SAP) for the Randomized Controlled Trial of an Internet-based Self-guided Hand Exercise Program to Improve Hand Function in People with Systemic Sclerosis: the Scleroderma Patient-centered Intervention Network Hand Exercise Program (SPIN-HAND) Trial**

**ADMINISTRATIVE INFORMATION**

**Registered at:** <https://clinicaltrials.gov/ct2/show/study/NCT03419208>

**SAP Version 1 (04-05-2020)**

**Amendment 1 (20-05-2020)**

**SAP contributors**:

1. Brett Thombs (McGill University, Canada)
2. Linda Kwakkenbos (Radboud University, the Netherlands)
3. Brooke Levis (Keele University, UK)
4. Andrea Benedetti (McGill University, Canada)

This SAP was drafted by Drs Brett Thombs, Linda Kwakkenbos, and Brooke Levis. All other contributors reviewed and approved of the final version. Dr. Andrea Benedetti is the senior statistican responsible for the integrity of all analyses. Dr. Brett Thombs is the principal investigator and clinical lead.

The SPIN-HAND Trial is a pragmatic, two-arm parallel cmRCT with a 3:2 allocation ratio to an offer of access to the SPIN-HAND Program in addition to usual care or to usual care alone that will be conducted using the SPIN Cohort. The primary outcome analysis will compare Cochin Hand Function Scale (CHFS) scores between participants randomly assigned to SPIN-HAND versus care as usual at 3 months post-randomization. Secondary outcomes will include CHFS score 6-, 12- and 24-months post-randomization and patient-reported functional health outcomes measured with the Patient Reported Outcomes Measurement Information System (PROMIS-29) profile version 2.0 and the EuroQoL-5D-5L at 3-, 6-, 12- and 24-months months post-randomization. Additionally, the Client Satisfaction Questionnaire-8 (CSQ-8) will be administered to intervention arm participants to assess program satisfaction.

1. Data will be checked for completeness and cleaned
2. Descriptive Analysis:

- By arm (SPIN-HAND vs Usual Care), we will describe demographic and disease characteristics (see draft Table).
- Participant reported satisfaction with the SPIN-HAND exercise program at 3 months post-randomization. The Client Satisfaction Questionnaire (CSQ-8) is an 8-item questionnaire evaluating user satisfaction. Items are scored on a 4-point Likert scale. Total scores range from 8 to 32, with higher scores indicating higher satisfaction with the service. Items have been modified slightly to refer to the SPIN-HAND program, as opposed to a generic service.
- Analysis of usage log data will be conducted to understand the uptake and use of the SPIN-HAND Program.

| **Variable** | **SPIN-HAND**  **N =** | **Usual Care**  **N =** |
| --- | --- | --- |
| **Demographic** |  |  |
| Age in years, mean (SD) |  |  |
| Female sex, N (%) |  |  |
| Education in years, mean (SD) |  |  |
| Married or living as married, N (%) |  |  |
| Race/ethnicity, N (%) |  |  |
| White |  |  |
| Black |  |  |
| Other |  |  |
| Country, N (%) |  |  |
| Canada |  |  |
| United States |  |  |
| United Kingdom |  |  |
| **Disease characteristics*** |  |  |
| Time since onset first non-Raynaud’s symptom or sign in years, mean (SD) |  |  |
| Time since onset Raynaud’s in years, mean (SD) |  |  |
| Time since diagnosis in years, mean (SD) |  |  |
| Diffuse disease subtype, N (%) |  |  |
| Modified Rodnan Skin Score, mean (SD) |  |  |
| Small joints contractures, N (% positive) |  |  |
| Large joint contractures, N (% positive) |  |  |
| Tendon friction rubs, N (% positive) |  |  |

*Disease characteristics were recorded at time of enrolment in the SPIN Cohort

1. Trial outcomes will be assessed immediately pre-randomization and at 3-months, 6-, 12- and 24-months post-randomization via the SPIN Cohort for both patients randomized to be offered the intervention and patients who receive usual care only.

All analyses will be conducted in R, and will be 2-sided, using an alpha value of 0.05. There will be no attempt to adjust for the multiplicity of secondary outcomes.

For the primary outcome analysis, we will use an intent-to-treat approach that compares CHFS scores between all patients randomized to be offered versus not offered the intervention 3-months post-randomization. The 18-item CHFS measures ability to perform daily hand-related activities (e.g., kitchen, dressing oneself, hygiene, writing/typing). Items are scored on a 0-5 Likert scale (0=*without difficulty; 5=impossible*). Higher scores indicate less functionality.

Intervention effects will be estimated using a generalized linear mixed model, adjusted for baseline CHFS scores, sex, age, disease duration, and diffuse versus limited SSc status as fixed effects. To account for the different enrollment centres, we will fit a random intercept for each site. We will generate 20 imputed datasets, using 15 cycles per dataset. Variables in the mice procedure will include: center of enrolment, intervention arm, consent, measures of all primary and secondary outcomes at all three timepoints, age, sex, subtype, years since onset of the first non-Raynaud’s phenomenon symptom, presence of small joint contractures, patient-reported severity of Raynaud’s phenomenon symptoms (0-10 scale) and digital ulcers (0-10 scale) at baseline. Pooled standard errors and confidence intervals will be estimated using Rubin’s rules.

1. Analysis of secondary outcome variables and time points (3-, 6-, 12- and 24-months post-randomization) will be done similar to the primary analysis. Secondary outcome measures include:
   1. The Patient Reported Outcomes Measurement Information System (PROMIS-29) profile version 2.0. The PROMIS-29 measures eight domains of health status with 4 items for each of 7 domains (physical function, anxiety, depression, fatigue, sleep disturbance, social roles and activities, pain interference) plus a single item for pain intensity.
   2. The EuroQoL-5D-5L is a 5-item standardized questionnaire that measures 5 dimensions (mobility, self-care, usual activities, pain/discomfort, and anxiety/depression). The items are rated from 1 (no problems) to 3 (extreme problems). Total scores reflect overall HRQL.
2. In addition to ITT analyses, we will use complier-average causal effect (CACE) analysis to estimate effects among patients who accept the intervention offer compared to similar patients in the usual care group. We will use an instrumental variable approach to inflate intent-to-treat effects from the primary analysis models by the inverse probability of compliance among participants in the intervention arm. 95% CIs will be constructed via bootstrap with resampling.

**Amendments to SAP (2020-05-20)**

*Removal of the EQ-5D:* Initially, the EuroQoL-5D-5L was specified as a secondary outcome for the SPIN-HAND trial to compute quality-adjusted life years for economic analyses. The EuroQol-5D, a 5-item standardized questionnaire, measures 5 dimensions (mobility, self-care, usual activities, pain/discomfort, and anxiety/depression). The items are rated from 1 (no problems) to 3 (extreme problems). Total scores reflect overall HRQL. Since items of the EuroQoL-5D-5L do not align with SPIN-HAND Program targets, it was not analyzed.

*Removal of 12- and 24-months time point:* The original trial protocol included analyses of 12- and 24-months post-randomization. Because we did not find evidence of improved hand function at 3 months or 6 months post-randomization and given the low uptake and use of the SPIN-HAND Program it was not plausible that there would be delayed effects. Therefore, we did not conduct planned analyses at 12- and 24-months post-randomization.

**eTable1: Trial outcomes: complete cases and estimated complier-average causal effect with complete cases**

|  |  | **Complete case analysis** | **Average complier effect with complete cases only** |
| --- | --- | --- | --- |
|  | **N** | **Difference (95% CI)** | **Difference (95% CI)** |
| **Primary Outcome (post-intervention)** |  |  |  |
| CHFS | 320 | -0.97 (-2.60 to 0.65) | -1.60 (-4.44 to 1.27) |
| **Secondary Outcomes (post-intervention)** |  |  |  |
| PROMIS-29v2 Physical function | 320 | 0.23 (-0.68 to 1.14) | 0.38 (-0.99 to 1.91) |
| PROMIS-29v2 Anxiety | 319 | 0.27 (-1.29 to 1.65) | 0.45 (-1.96 to 2.86) |
| PROMIS-29v2 Depression | 319 | -0.63 (-2.13 to 0.87) | -1.03 (-3.20 to 1.61) |
| PROMIS-29v2 Fatigue | 319 | -0.32 (-1.86 to 1.22) | -0.53 (-3.01 to 1.99) |
| PROMIS-29v2 Sleep disturbance | 317 | 0.38 (-1.07 to 1.83) | 0.62 (-1.76 to 2.90) |
| PROMIS-29v2 Social roles and activities | 319 | -0.63 (-1.86 to 0.61) | -1.02 (-2.94 to 1.09) |
| PROMIS-29v2 Pain interference | 318 | -0.01 (-1.42 to 1.41) | -0.01 (-2.41 to 2.51) |
| PROMIS-29v2 Single item for pain intensity | 317 | 0.16 (-0.23 to 0.56) | 0.27 (-0.32 to 1.06) |
| **Secondary Outcomes (6 months post-randomization):** |  |  |  |
| CHFS | 321 | -0.97 (-2.60 to 0.65) | -1.60 (-4.44 to 1.27) |
| PROMIS-29v2 Physical function | 325 | 0.23 (-0.68 to 1.14) | 0.38 (-0.99 to 1.91) |
| PROMIS-29v2 Anxiety | 325 | 0.27 (-1.29 to 1.65) | 0.45 (-1.96 to 2.86) |
| PROMIS-29v2 Depression | 325 | -0.63 (-2.13 to 0.87) | -1.03 (-3.20 to 1.61) |
| PROMIS-29v2 Fatigue | 324 | -0.32 (-1.86 to 1.22) | -0.53 (-3.01 to 1.99) |
| PROMIS-29v2 Sleep disturbance | 325 | 0.38 (-1.07 to 1.83) | 0.62 (-1.76 to 2.90) |
| PROMIS-29v2 Social roles and activities | 325 | -0.63 (-1.86 to 0.61) | -1.02 (-2.94 to 1.09) |
| PROMIS-29v2 Pain interference | 324 | -0.01 (-1.42 to 1.41) | -0.01 (-2.41 to 2.51) |
| PROMIS-29v2 Single item for pain intensity | 323 | 0.16 (-0.23 to 0.56) | 0.27 (-0.32 to 1.06) |

Results presented are score differences between the SPIN-HAND and usual care arms
